# Beneficial Effects of Pterocarpan-High Soybean Leaf Extract on Metabolic Syndrome in Overweight and Obese Korean Subjects: Randomized Controlled Trial

**DOI:** 10.3390/nu8110734

**Published:** 2016-11-18

**Authors:** Ri Ryu, Tae-Sook Jeong, Ye Jin Kim, Ji-Young Choi, Su-Jung Cho, Eun-Young Kwon, Un Ju Jung, Hyeon-Seon Ji, Dong-Ha Shin, Myung-Sook Choi

**Affiliations:** 1 Department of Food Science and Nutrition, Kyungpook National University, 80 Daehakro, Bukgu, Daegu 41566, Korea; sangsang0119@gmail.com (R.R.); freewilly59@hanmail.net (Y.J.K.); 2 Center for Food and Nutritional Genomics Research, Kyungpook National University, Daegu 702-701, Korea; jyjy31@hanmail.net (J.-Y.C.); chocrystalhihi@hanmail.net (S.-J.C.); savagegarden01@hanmail.net (E.-Y.K.); 3 Industrial Bio-Materials Research Center, Korea Research Institute of Bioscience and Biotechnology, Daejeon 305-806, Korea; tsjeong@kribb.re.kr (T.-S.J.); gustjs1693@naver.com (H.-S.J.); 4 Department of Food Science and Nutrition, Pukyong National University, Busan 608-737, Korea; jungunju@naver.com; 5 College of Pharmacy, Chungnam National University, Daejeon 305-764, Korea; 6 Insect Biotech Co., Ltd., Daejeon 305-811, Korea; dhshin@insectbiotech.co.kr

**Keywords:** inflammation, metabolic syndrome, mRNA sequencing, soybean leaf, PBMCs

## Abstract

Pterocarpans are known to have antifungal and anti-inflammatory properties. However, little is known about the changes in transcriptional profiles in response to a pterocarpan-high soybean leaf extract (PT). Therefore, this study investigated the effects of PT on blood glucose and lipid levels, as well as on the inflammation-related gene expression based on a peripheral blood mononuclear cells (PBMCs) mRNA sequencing analysis in Korean overweight and obese subjects with mild metabolic syndrome. The participants were randomly assigned to two groups and were administered either placebo (starch, 3 g/day) or PT (2 g/day) for 12 weeks. The PT intervention did not change body weight, body fat percentage and body mass index (BMI). However, PT significantly decreased the glycosylated hemoglobin (HbA1c), plasma glucose, free fatty acid, total cholesterol, and non-HDL cholesterol levels after 12 weeks. Furthermore, PT supplementation significantly lowered the homeostatic index of insulin resistance, as well as the plasma levels of inflammatory markers. Finally, the mRNA sequencing analysis revealed that PT downregulated genes related to immune responses. PT supplementation is beneficial for the improvement of metabolic syndrome by altering the fasting blood and plasma glucose, HbA1c, plasma lipid levels and inflammation-related gene expression in PBMCs.

## 1. Introduction

Obesity increases the risk of metabolic abnormalities associated with insulin resistance, hyperglycemia, type 2 diabetes, and dyslipidemia. The increasing prevalence of obesity and obesity-related diseases increases healthcare costs and also downgrades the quality of life [1]. Obesity, as well as excess fat intake, leads to the accumulation of body fat mass. In particular, central obesity or abnormally high deposition of visceral adipose tissue is considered a risk factor for metabolic complications [2]. In obesity, excess triglycerides promote the lipolysis and release of free fatty acids (FFA), which results in the dysfunction of insulin action and in insulin resistance. Elevated levels of circulating FFA can disrupt the insulin signaling in peripheral tissues and decrease the insulin sensitivity [3]. In addition, high levels of plasma FFA increase the expression of several pro-inflammatory cytokines, such as tumor necrosis factor alpha (TNF-α), interleukin 6 (IL-6), and monocyte chemotactic protein-1 (MCP-1) [4]. It should be noted, however, that obesity is influenced not only by the energy imbalance but also by many other factors, including environmental, behavioral, genetic, and metabolic predisposition [5,6,7,8].

Peripheral blood mononuclear cells (PBMCs), composed of cells, including lymphocytes, monocytes, and dendritic cells, can be easily obtained from blood and have been used as a tool in nutrition studies [9] and as a common target of immunological studies in animals and humans [10]. In addition, the transcriptome or gene expression analysis in PBMCs can reveal information on the lipid metabolism in obesity-associated organs, adipose tissue, and the liver [11].

Recently, many functional food sources have been used to prevent or improve metabolic diseases. Among those, soybean has been documented to be associated with reduction in the risk for cancers [12,13], body weight and cholesterol level [14] and it contains bioactive components, isoflavones (daidzin, genistin, and malonylgenistin), that can prevent obesity, breast cancer, and cardiovascular disease [15,16]. However, soy leaves contain pterocarpan and kaempferol glycosides, while they contain small amounts of isoflavones [17,18]. A kaempferol glycoside (KG)-rich fraction from unripe soybean leaf attenuated blood glucose and hepatic lipid levels in KK-A^y^ mice [19]. In particular, pterocarpans such as coumesterol, glyceofuran, glyceollin III suppressed the oxidation of low-density lipoprotein (LDL) [20], and have been reported to have cancer prevention and anti-inflammatory effects [21,22,23]. In addition, glyceollins (I–III), prenylated pterocarpan have known antiestrogenic effects in vivo [24,25]. In a previous study, soy leaf exhibited non-HDL-cholesterol lowering effects in hamsters [26]—as well as ethyl acetate extracts of soybean leaves, including pterocarpan—ameliorated the insulin sensitivity and improved the plasma glucose levels in high-fat diet (HFD)-induced type 2 diabetic mice [27]. We have also reported that the supplementation with soybean leaves (2 g/day) is beneficial for lowering the fasting blood glucose and plasma triglyceride levels in adult subjects with prediabetes [28], while another study found that soybean leaves supplementation (2 g/day) failed to promote weight-loss [29]. However, little is known about the exact mechanisms associated with the anti-metabolic disease action of a pterocarpan-high soybean leaf extract (PT), especially in terms of integrating transcriptional profiles and phenotypic biomarkers from human subjects. 

In this study, we investigated the effects of PT on metabolic parameters and PBMCs transcriptional profiles in overweight and obese subjects. This is the first study that reports the metabolic effect of PT and the related gene expression changes in PBMCs using mRNA sequencing analysis in overweight and obese subjects with mild metabolic syndrome. 

## 2. Materials and Methods 

### 2.1. Subjects

Volunteers (35–65 years old) were recruited from Daegu and among employees of Kyungpook National University in the Republic of Korea from December 2013 to February 2014. Fifty subjects (27 kg·m^−2^ ≥ BMI ≥ 23 kg·m^−2^) with mild metabolic syndrome were enrolled in this study. According to the recommendations of World Health Organization (Asia-Pacific Region), we applied an intermediate cut-off point of BMI as 23 kg·m^−2^, because health risk is higher in Asian populations at a lower BMI [30]. Eligibility criteria for participants included three or more of these traits: waist circumference ≥90 cm (male) and ≥80 cm (female); triglyceride level ≥150 mg/dL; high-density lipoprotein (HDL) cholesterol <40 mg/dL and <50 mg/dL (female); fasting blood glucose (FBG) ≥95 mg/dL. Excluded were pregnant women, type 1 diabetics, those treated with insulin and any drugs to control blood glucose, blood lipids, and body weight, and those using functional food products that might affect the results of this study. The trial was performed in accordance with the Declaration of Helsinki, and all subjects provided written informed consent prior to participating in this study. Experiments were performed according to the guidelines of the Ethics Committee of Kyungpook National University (Approval number KNU 2013-0018). The study protocol were registered in The Clinical Research Information Service (KCT 0002056). 

### 2.2. Preparation of Pterocarpan-High Soybean Leaf Extract 

Soybeans, *Glycine max* (L.) Merr., were cultivated in Jeungpyeong county, Chungcheongbuk-do, Korea, for four months. Soybean leaves were harvested in September 2013. Air-dried leaves (50 kg) were extracted with 500 L of 95% ethanol for two days at 25–30 °C. A pterocarpan-high soybean leaf extract (PT) was concentrated in vacuum and lyophilized to yield a dark brown powder (8.7 kg). The PT dissolved in methanol was filtered through a 0.45-μm poly (tetrafluoroethylene) filter (Whatman International, Ltd., Maidstone, UK) for HPLC analysis [27]. Coumestrol, which was obtained from Sigma-Aldrich (St. Louis, MO, USA), and phaseol, which was isolated from the PT [18], were used as external standards for HPLC analysis. The contents of coumestrol and phaseol in the PT were measured using HPLC–diode-array detection, with standard curves provided (Figure S1). The contents of coumestrol and phaseol were 10.85 ± 0.26 and 5.90 ± 0.11 μg/mg of PT, respectively.

The total flavonoid contents of the extracts were measured using a modified colorimetric method [31] and were expressed as mg quercetin equivalents (QE)/g extract. The total phenolic contents of the extracts were measured using a modified version of the Folin–Ciocalteu method [31] and were expressed as mg gallic acid equivalent (GAE)/g extract. The PT included 136.7 ± 0.0 (GAE)/g extract total flavonoids and 77.3 ± 0.2 (QE)/g extract total phenolic (Table S1). The PT capsules were produced by in Insect Biotech Co., Ltd. (Daejeon, Korea).

### 2.3. Study Design

This study was a randomized double-blinded, placebo-controlled, parallel trial to evaluate the glucose-, and lipid-lowering effects of PT in overweight and obese subjects with mild metabolic syndrome. 

The random allocation sequence was created using computer random numbers. At randomization, all subjects were randomly assigned in 1:1 ratio, two nutritional intervention groups: placebo (*n* = 25) and PT (*n* = 25). The mechanism used for allocation concealment was sequentially numbered containers by an independent laboratory researcher, and participants were kept blinded to the sequence and randomization until the end of the study. The capsules containing PT and placebo were no different, including undistinguishable size and color. Each participant received the capsules in the prepacked white plastic containers. The primary outcomes were fasting blood glucose, hemoglobin A1c, plasma glucose, and secondary outcomes were the homeostatic model assessment of insulin resistance, plasma lipid levels, and plasma cytokines-related metabolic syndrome and inflammation. 

The subjects in the placebo group consumed six capsules containing starch (3 g per day), and those in the PT group consumed PT (2 g per day) in the morning, afternoon, and evening daily for 12 weeks. All participants were instructed to maintain their routine food intake and physical activity during the study. During the study period, we monitored the compliance of the subjects with the nutritional intervention and capsule consumption every week by telephone. 

### 2.4. Anthropometric and Biochemical Analyses

At baseline and after the 4-, 8-, and 12-week nutritional intervention, the subjects attended the Science Research Center laboratory at Kyungpook National University between the hours of 07:00 and 11:00 a.m. after a 12-h overnight fast for anthropometric and physiological measurements. The waist circumference, hip circumference, blood pressure, fasting blood glucose (FBG), glycosylated hemoglobin (HbA1c), and lipids were determined at baseline and after the 12-week nutritional intervention. The body mass index (BMI), height, weight, and body composition were measured using an X-Scan plus II body composition analyzer (Jawon Medical Company, Daejeon, Korea). The waist and hip circumferences were measured with an anthropometric tape. The waist circumference was measured at the minimum circumference between the iliac crest and rib cage, and the hip circumference was measured at the maximum width over the greater trochanters. The waist-to-hip ratio (WHR) was then calculated by dividing the waist measurement by the hip measurement. The FBG, HbA1c, and blood pressure were measured using a glucose analyzer (LifeScan Inc., Milpitas, CA, USA), an HbA1c analyzer (Micormat™ Hemoglobin A1c Test, Bio-Rad, Hercules, CA, USA), and an automatic blood pressure (BP) monitor (Omron, Kyoto, Japan), respectively. In addition, blood samples were drawn into heparin-coated tubes and then centrifuged at 1000× *g* for 15 min at 4 °C for plasma assays. Dietary intake was recorded using 24-h dietary recalls for each subject before and during the nutritional intervention trial. Nutritional analysis was performed using the CAN-Pro 3.0 software (The Korean Nutrition Society, Seoul, Korea), which provides a comprehensive database for the nutritional content of general foods and specialty Korean foods.

### 2.5. Plasma Lipid Analyses

Plasma lipid concentrations were determined using commercially available kits for total cholesterol, triglycerides, HDL cholesterol (Asan Pharm. Co., Seoul, Korea), and free fatty acids (FFA) (Wako Chemicals, Richmond, VA, USA). The LDL cholesterol level was calculated using the Friedewald formula [32]: [total cholesterol-HDL cholesterol-(triglycerides/5)]. The non-HDL cholesterol level was calculated as follows: HDL cholesterol-total cholesterol. The atherogenic index (AI) was calculated as follows: (total cholesterol-HDL cholesterol)/HDL cholesterol.

### 2.6. Biochemical Analyses

Plasma adiponectin and leptin levels were determined using a commercial quantitative enzyme-linked immunosorbent assay (ELISA) kits (R&D Systems, Minneapolis, MN, USA). The levels of plasma insulin, plasminogen activator inhibitor-1 (PAI-1), and cytokines [interleukin 6 (IL-6), monocyte chemotactic protein-1 (MCP-1), and tumor necrosis factor alpha (TNF-α)] were determined using multiplex detection kits from Bio-Rad. All samples were assayed in duplicate and analyzed with a Luminex 200 LabMAP system (Luminex, Austin, TX, USA). Data analyses were carried out using the Bio-Plex Manager software version 4.1.1 (Bio-Rad). Plasma aspartate aminotransferase (AST) and alanine aminotransferase (ALT) were determined using enzymatic kits (Asan Pharm. Co.). The index of insulin resistance was calculated according to the homeostatic model assessment of insulin resistance (HOMA-IR) formula [33]: [fasting glucose (mmol·L^−1^) × fasting insulin (mU·L^−1^)]/22.5.

### 2.7. Isolation of Peripheral Blood Mononuclear Cells and Extraction of RNA

Peripheral blood mononuclear cells (PBMCs) were isolated from the heparin-treated blood samples by density gradient centrifugation with the Ficoll–Paque reagent (GE Healthcare, Piscataway, NJ, USA) and were used for total RNA extraction. Total RNA was extracted using the TRIzol reagent (Invitrogen, Carlsbad, CA, USA) according to the manufacturer’s instructions. The purity and integrity of the isolated RNA were evaluated using an Agilent 2100 Bioanalyzer (Agilent Technologies, Santa Clara, CA, USA).

### 2.8. mRNA Sequencing Analysis

For mRNA sequencing analysis, PBMCs were collected from three subjects randomly selected from the PT groups at baseline and 12 weeks. The mRNA in the total RNA was converted into a library of template molecules suitable for subsequent cluster generation using the reagents provided in the Illumina^®^ TruSeq™ RNA Sample Preparation Kit (Illumina, Inc., San Diego, CA, USA). The first step in the workflow involved the purification of poly-A-containing mRNA molecules using poly-T oligo-attached magnetic beads. Following the purification, the mRNA was fragmented into small pieces using divalent cations at an elevated temperature. The cleaved RNA fragments were reverse-transcribed into first-strand cDNA using a reverse transcriptase and random primers. This was followed by the second-strand cDNA synthesis using DNA polymerase I and RNase H. These cDNA fragments then underwent an end repair process, the addition of a single “A” base, and ligation of the adapters. The products were then purified and enriched by polymerase chain reaction (PCR) to create the final cDNA library.

### 2.9. Preprocessing of RNA-seq Data

Quality control of the reads was performed using FastQC v. 0.10.0 (Babraham Bioinformatics, Cambridge, UK). The remaining reads were mapped onto a reference genome using the aligner software, TopHat version 1.3.3 (Johns Hopkins University, Baltimore, MD, USA). Then, the transcripts were assembled in Cufflink v. 2.0.2 using the gene annotation database of the TopHat Aligner. After the assembly, expression levels were measured in fragments per kilobase of transcript per million mapped reads (FPKM). The RNA-seq data has been submitted to the publicly available NCBI’s Gene Expression Omnibus Database (http://www.ncbi.nlm.nih.gov/geo/) [34], accession number GSE80714.

### 2.10. Differential Transcriptome and Pathway Analysis

Differentially expressed genes were identified based on both the *p*-value threshold of less than 0.05 and a 1.5-fold change, and the Kyoto Encyclopedia of Genes and Genomes (KEGG) pathways (www.genome.jp/kegg) were used for analyzing gene functions.

### 2.11. Real-Time Quantitative PCR

Total RNA was reverse-transcribed into cDNA using the QuantiTect reverse transcription kit (QIAGEN Gmblh, Hilden, Germany), and mRNA expression was quantified by real-time quantitative PCR using the SYBR green PCR kit (QIAGEN Gmblh) and a CFX96TM real-time system (Bio-Rad). Primers were designed to detect Phosphoinositide-dependent protein kinase-1 (PDPK-1, 5170), CC chemokine receptor 4 (CCR4, 1233), CC chemokine receptor 6 (CCR6, 1235) and CC chemokine ligand 4-like 2 (CCL4L2, 9560). The amplification was performed as follows: 10 min at 90 °C, 15 s at 95 °C, and 60 s at 60 °C, for a total of 40 cycles. The Ct values were normalized to corresponding glyceraldehyde 3-phosphate dehydrogenase (GAPDH) values, and the relative gene expression was calculated using the 2^−ΔΔCt^ method [35]. 

### 2.12. Statistical Analysis

The sample size for this trial was determined between two independent sample means (two tailed *t*-test, *p* < 0.05), a minimum of 20 participants per group was required. All data are presented as the mean ± standard error of the mean (SE). Statistical analysis was performed using SPSS Statistics, version 21 (IBM, Chicago, IL, USA). Significant changes within the groups between the baseline and 12-week values were assessed using a paired Student’s *t*-test. The differences between the groups were evaluated by the General Linear Model with two-way repeated-measures ANOVA and baseline value, age, gender and BMI as covariates. Statistically significant differences were accepted as *p* < 0.05. 

## 3. Results

### 3.1. Baseline Clinical Characteristics

Fifty subjects were enrolled in this study. Among the enrolled subjects, one of the subjects dropped out of this study for personal reasons. Therefore, 49 subjects completed the trial [placebo (*n* = 25) and PT (*n* = 24)] from March to June 2014. Five subjects were excluded because of poor compliance [consuming <80% of the instructed amount, placebo (*n* = 2) and PT (*n* = 3)]. Serious adverse effects were not reported by the subjects consuming the PT or placebo supplements. In both males and females, there were no significant differences in the age, body weight, BMI, waist-to-hip ratio (WHR), blood pressure, and fasting blood glucose (FBG) levels between the groups before the trial (Table 1). In addition, all subjects were overweight or obese (27 kg·m^−2^ ≥ BMI ≥ 23 kg·m^−2^) [30,36].

### 3.2. Nutrient Intake

Analysis of the participants’ 24-h dietary recalls indicated no significant differences in the energy and nutrient intake between the groups at baseline or after 12 weeks of the supplementation (Table 2). 

### 3.3. Anthropometric Parameters and Blood Pressure

After 12 weeks of supplementation with PT, the systolic blood pressure (*p* = 0.045) was significantly lowered relative to the baseline values. However, there were no significant interaction effects (time × group) in the body weight, BMI, body fat percentage, WHR, and blood pressure versus the baseline values between the groups (Table 3). 

### 3.4. Fasting Blood Glucose, HbA1c, Insulin, Plasma Glucose Levels and Homeostatic Model Assessment of Insulin Resistance

Two-way repeated-measures ANOVA showed significant interaction effects on FBG (*p* = 0.017) and HbA1c (*p* = 0.023), but there was no significant difference in the FBG concentration in the PT group after the trial (Table 4). After the 12-week trial, the plasma glucose level (*p* = 0.000) and the homeostatic model assessment of insulin resistance (HOMA-IR) index (*p* = 0.000) were significantly decreased in the PT group (Table 4). Furthermore, there were significant differences in the plasma glucose (*p* = 0.001) and HOMA-IR (*p* = 0.024) levels between the groups. PT significantly reduced the plasma insulin concentration (*p* = 0.012) after 12 weeks, although there was no significant interaction effect (time × group) (Table 4). 

### 3.5. Aspartate Transaminase and Alanine Transaminase Activities in Plasma

The plasma aspartate transaminase (AST) and alanine transaminase (ALT) activities were not different in the placebo and PT group during the trial, nor between the groups (Table 5). 

### 3.6. Plasma Lipid Levels

At baseline, the plasma triglyceride, total cholesterol, FFA, HDL cholesterol, non-HDL cholesterol, LDL cholesterol levels, and the atherogenic index (AI) were not significantly different between the two groups (Table 6). After 12 weeks, FFA (*p* = 0.000), total cholesterol (*p* = 0.020), and non-HDL cholesterol (*p* = 0.014) levels were significantly decreased in the PT group (Table 6). In addition, two-way repeated-measures ANOVA showed significant effects on the plasma FFA (*p* = 0.000), and non-HDL cholesterol (*p* = 0.014) levels between the groups (Table 6). Meanwhile, there were no significant interaction effects (time × group) in the plasma triglyceride, HDL cholesterol, LDL cholesterol, and AI values at interaction between the groups (Table 6). 

### 3.7. Plasma Adipokine and Cytokine Levels 

At baseline, no significant differences were observed in the plasma TNF-α, plasminogen activator inhibitor-1 (PAI-1), MCP-1, and IL-6 levels between the groups (Table 7). After the 12-week intervention, the PT supplement lowered the levels of TNF-α (*p* = 0.046), PAI-1 (*p* = 0.015), and IL-6 (*p* = 0.030), whereas the MCP-1 level was not changed (Table 7). Furthermore, there were significant interactions in plasma TNF-α (*p* = 0.031) and PAI-1 (*p* = 0.044) between the groups (Table 7). The plasma adiponectin and leptin levels showed no statistically significant differences in the placebo and PT group after the 12 weeks, nor was interaction between the groups affected (Table 5). 

### 3.8. Gene Expression Profiles of PBMCs Based on mRNA Sequencing Analysis

The PBMCs mRNA sequencing analysis identified genes that were differentially expressed before and after the PT supplementation. This comparison showed that 37 genes were upregulated and 128 genes were downregulated by the PT supplement. 

**Figure 1:**
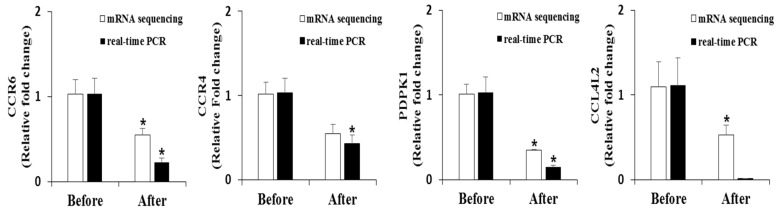
RT–qPCR validation of mRNA sequencing results for peripheral blood mononuclear cells. Fold changes in mRNA expression after the supplementation of pterocarpan-high soybean leaf extract relative to the baseline levels. The mRNA sequencing and RT–qPCR data are shown as the mean ± S.E. * *p* < 0.05. PDPK-1, phosphoinositide-dependent protein kinase-1; CCR, CC chemokine receptor; CCL4L2, CC chemokine ligand 4-like 2.

To elucidate the functional differences in the PBMCs transcriptome caused by the PT supplement, we used the KEGG pathways mapper tool for the genes that were differentially expressed after the 12-week intervention. In the PT group, the differentially expressed gene-enriched pathways included chemokine signaling pathways, cytokine–cytokine receptor interactions, nuclear factor kappa B signaling, fatty acid metabolism, peroxisome proliferator-activated receptor signaling, and insulin signaling pathways (Table 8). In addition, genes involved in these pathways were associated with immune responses. Selected results of the mRNA sequencing analyses were confirmed by real-time–quantitative polymerase chain reaction (real-time–qPCR) (Figure 1). 

## 4. Discussion

In a previous study, PT ameliorated the insulin sensitivity and β-cell dysfunction in type 2 diabetic mice [27]. However, no clinical trial has been conducted to examine the anti-metabolic disorder effects with the transcriptome analysis in PT-supplemented subjects. Therefore, in the current study, we evaluated the effects of PT on diabetes-associated phenotype markers, plasma lipid, and plasma inflammatory cytokine levels, as well as on PBMCs transcriptional responses, in subjects with mild metabolic syndrome and compared the results with those obtained for placebo-supplemented subjects.

Previous studies have shown that supplementation with pterocarpan (coumesterol and phaseol) -rich soy leaf reduced body weight and lipid accumulation by regulating adipogenic transcription factors in obese mice-fed HFD [37]. Additionally, Choi et al. [28] have demonstrated that supplementation with 70% ethanol extracts of soybean leaf has body fat-lowering effect in prediabetic patients, while Kim et al. [29] reported that 95% ethanol extracts of soybean leaf had a minimal effect on % body fat in overweight subjects. However, in the present study, the body weight, BMI, and body fat content were not significantly different compared to baseline after 12 weeks. Thus, our previous [29] and present studies suggest that the body fat-lowering effect of soybean leaf extracts may be associated with solvent and experimental conditions. 

The HbA1c level is a useful parameter for monitoring diabetes and glucose tolerance since HbA1c reflects the degree of blood glucose regulation over two to three months [38]. In the current study, the supplementation with PT led to a significant decrease in the blood HbA1c and FBG levels after 12-week intervention. Thus, the present study indicates that the supplementation with PT can regulate long-term hyperglycemia by decreasing the blood HbA1c and FBG levels. Our previous study has demonstrated that the soybean leaf extract (2 g per day) supplementation for 12 weeks significantly decreased the blood HbA1c and FBG levels compared to the baseline [28]. Similarly, Soy leaf extract containing KG improved diabetes-associated phenotypes and glucose homeostasis in *db/db* mice [39]. In addition, the plasma insulin level was significantly lowered after the PT supplementation. Thus, we evaluated the HOMA-IR, a useful index for assessing insulin resistance, insulin sensitivity, and β-cell function. The PT supplement significantly decreased the HOMA-IR value after the trial, as well as resulting in significant differences in the HOMA-IR value between the groups. The HOMA-IR lowering effect of the soybean leaf extracts were consistent between our previous human [28,29] and current studies. Taken together, these results suggest that the PT supplementation can provide improvements in hyperglycemic subjects. 

The present study also demonstrated that the supplementation with PT significantly decreased the plasma total cholesterol, non-HDL cholesterol, and FFA levels; the significant interaction effect of FFA was also observed between the groups. Non-HDL cholesterol is regarded as an important indicator of cardiovascular risk [40]. In addition, soy leaves have been reported to reduce non-HDL cholesterol, although serum total cholesterol showed a trend of lowering effects in hamsters [26]. An increase in the plasma FFA level is associated with metabolic disorders and insulin resistance, while FFA reduction partially leads to improved glucose metabolism through a decrease of gluconeogenesis and glycogenolysis [41,42,43]. Taken together, these results support that PT supplementation contributes to improving hyperglycemia and insulin sensitivity by decreasing the plasma FFA concentration. 

Obesity is linked to chronic low-grade inflammation and elevates the production of proinflammatory adipokines, which can cause the development of type 2 diabetes and obesity-related comorbidities [44,45]. In general, proinflammatory plasma markers such as TNF-α, IL-6, and MCP-1 are present in an early inflammatory stage of obesity. On the other hand, macrophage inflammatory protein-1β (MIP-1β) levels increase when obesity is well established [46]. TNF-α is produced in an insulin-resistant state and reduces the expression level of plasma adiponectin [47]. PAI-1 is not only associated with thrombosis and fibrosis but also with obesity, metabolic syndrome and insulin resistance [48]. Furthermore, there is a positive correlation between the levels of TNF-α, which has been reported to be a potent PAI-1 inducer, and PAI-1 [49]. In this trial, the plasma TNF-α, IL-6 and PAI-1 levels were significantly decreased in the PT group after the 12 weeks of supplementation, although the interaction effect of time and group showed only the TNF-α level between the groups. In addition, a previous study showed that pterocarpan-enriched soy leaf supplementation led to inhibiting the gene expression of TNF-α and IL-6 in the white adipose tissue in type 2 diabetic mice [27]. Taken together, these observations indicate that the decreased TNF-α level may be induced by improving the plasma PAI-1 level. Moreover, decreased cytokine levels are partially linked to enhanced insulin sensitivity and glucose homeostasis.

Phosphoinositide-dependent protein kinase-1 (PDPK-1) is the downstream kinase of phosphatidylinositol-3 kinase and is stimulated by insulin. It also targets protein kinase B, the main effector of PDPK-1 [50]. In addition, Tawaramoto et al. [51] reported that the knockout of vascular endothelial PDPK-1 improved obesity and insulin sensitivity in HFD-fed knockout mice by reducing the visceral fat accumulation compared to that in the wild-type mice. Our mRNA sequencing analysis in PBMCs revealed downregulated mRNA expression of PDPK-1 in PT-supplemented subjects, possibly owing to a decreased insulin level. 

Chemokines are considered therapeutic targets for inflammatory diseases [52]. In the current study, the mRNA expression of CC chemokine receptor (CCR) 6, CCR4, and CC chemokine ligand 4-like 2 (CCL4L2), which is similar to CCL4, was downregulated. In obesity, inflammatory mediators such as IL-1β, TNF-α, and interferon gamma can elevate the CCR6 expression level [53]. In addition, CCR4 and CCR6 are more expressed in type 2 diabetes patients than in non-diabetic persons [54]. CCL4, also known as MIP-1β, is expressed at two-fold higher levels in obese and metabolic syndrome subjects compared with those in healthy individuals [55]. Our data suggest that PT has the potential to regulate insulin resistance and metabolic syndrome by lowering the mRNA expression of PDPK-1, CCR4, CCR6, and CCL4L2 in PBMCs. 

There are some limitations of this trial. Participants of the present study were overweight or obese subjects with mild metabolic syndrome. However, the impact of the study would be higher when lean or different types of subjects are used to identify clear interpretation. 

## 5. Conclusions

In conclusion, the present study indicates that the supplementation with PT (2 g/day) can reduce the risk factors associated with diabetes and dyslipidemia in obese subjects with mild metabolic syndrome and simultaneously decrease the inflammatory-related gene expression in PBMCs.

## Figures and Tables

**Table 1:** Comparison of baseline clinical characteristics of subjects.

	Placebo		PT	
	Female	Male	Female	Male
***n***	19	6	19	5
**Age** (years)	47.68 ± 1.80	50.57 ± 2.72	50.53 ± 1.50	50.80 ± 3.43
**Body weight** (kg)	65.54 ± 1.13	74.22 ± 3.99	63.12 ± 1.47	78.18 ± 4.67
**BMI** (kg/m^2^)	26.11 ± 0.44	25.52 ± 0.84	25.24 ± 0.40	26.46 ± 1.00
**Systolic BP** (mmHg)	137.16 ± 3.22	144.67 ± 10.08	128.37 ± 3.83	146.00 ± 12.88
**Diastolic BP** (mmHg)	79.79 ± 1.72	85.17 ± 5.21	73.16 ± 2.79	86.80 ± 8.63
**WHR**	0.86 ± 0.01	0.91 ± 0.01	0.84 ± 0.01	0.89 ± 0.01
**FBG** (mg/dL)	109.17 ± 6.48	104.47 ± 4.35	113.67 ± 10.42	104.47 ± 4.35

Values are the mean ± SE. Placebo, starch; PT, pterocarpan-high soy leaf extract (2 g/day); BMI, body mass index; BP, blood pressure; WHR, waist-hip ratio; FBG, fasting blood glucose.

**Table 2:** Changes in nutrient intake.

	Placebo		PT	
	Before	After	Before	After
**Energy** (kcal/day)	1728.58 ± 63.52	1705.32 ± 47.26	1772.17 ± 76.46	1698.56 ± 58.23
**Protein** (g/day)	69.94 ± 3.19	73.99 ± 3.68	68.97 ± 4.49	68.31 ± 3.88
**Fat** (g/day)	43.46 ± 3.05	44.25 ± 3.65	49.42 ± 4.10	47.89 ± 4.57
**Carbohydrate** (g/day)	268.45 ± 12.18	259.87 ± 11.49	271.87 ± 12.65	252.02 ± 10.96
**Fiber** (g/day)	7.68 ± 0.54	7.61 ± 0.58	8.64 ± 0.66	7.78 ± 0.61
**Ca** (mg/day)	469.28 ± 49.24	54.36 ± 40.58	595.57 ± 64.03	469.89 ± 50.76
**P** (mg/day)	1018.68 ± 44.64	1054.29 ± 44.65	1064.23 ± 77.10	980.48 ± 68.70
**Fe** (mg/day)	13.67 ± 0.59	15.52 ± 0.95	14.66 ± 0.81	13.77 ± 1.23
**Vit. B1** (mg/day)	1.15 ± 0.06	1.23 ± 0.08	1.14 ± 0.10	1.20 ± 0.09
**Vit. B2** (mg/day)	1.08 ± 0.06	1.36 ± 0.06	1.09 ± 0.11	1.17 ± 0.21
**Vit. B6** (mg/day)	2.07 ± 0.12	2.34 ± 0.16	2.37 ± 0.19	2.19 ± 0.17
**Vit. C** (mg/day)	102.34 ± 10.77	120.26 ± 19.49	113.65 ± 17.53	108.34 ± 15.42
**folate** (μg/day)	261.98 ± 18.70	372.46 ± 66.01	278.38 ± 20.29	400.60 ± 99.63
**Vit. E** (mg/day)	11.81 ± 1.45	12.68 ± 1.30	16.10 ± 1.74	10.39 ± 1.26
**Cholesterol** (mg/day)	313.25 ± 48.50	330.26 ± 40.02	308.92 ± 45.48	274.47 ± 35.33

Values are the mean ± SE. Placebo, starch; PT, pterocarpan-high soy leaf extract (2 g/day).

**Table 3:** Effect of pterocarpan-high soy leaf extract supplementation on changes of BMI, BFP, WHR-related body measurements and blood pressure in subjects with metabolic syndrome.

	Placebo		PT		Time × Group, *p* Value ^†^
	Before	After	Before	After	
**Body weight** (kg)	67.79 ± 1.50	67.25 ± 1.56	66.09 ± 2.08	64.80 ± 2.23	0.174
**BMI** (kg/m^2^)	25.97 ± 0.39	25.65 ± 0.38	25.51 ± 0.40	25.15 ± 0.44	0.827
**BFP** (%)	32.08 ± 0.95	31.14 ± 0.87	32.41 ± 0.72	31.04 ± 0.77	0.122
**WHR**	0.87 ± 0.01	0.88 ± 0.01	0.85 ± 0.01	0.85 ± 0.01	0.174
**Systolic BP** (mmHg)	136.45 ± 2.77	128.77 ± 2.38	131.68 ± 3.12	126.00 ± 3.37 *	0.624
**Diastolic BP** (mmHg)	81.24 ± 1.83	77.12 ± 2.05	76.54 ± 2.73	72.13 ± 2.62	0.497

Values are the mean ± SE. Placebo, starch; PT, pterocarpan-high soy leaf extract (2 g/day); BMI, body mass index; BFP, body fat percentage; VFA, visceral fat area; WHR, waist-hip ratio; BP, blood pressure. * *p* < 0.05 paired *t*-test between before and after trial in each group; ^†^ *p* < 0.05 general linear model for repeated measures ANOVA.

**Table 4:** Effect of pterocarpan-high soy leaf extract supplementation on changes of FBG, HbA1c, plasma glucose, insulin concentrations and HOMA-IR in subjects with metabolic syndrome.

	Placebo		PT		Time × Group, *p* Value ^†^
	Before	After	Before	After	
**FBG** (mg/dL)	104.61 ± 3.77	103.83 ± 3.54	107.76 ± 4.62	99.14 ± 3.19	0.017
**HbA_1_c** (%)	5.56 ± 0.10	5.69 ± 0.11	5.60 ± 0.09	5.44 ± 0.10 *	0.023
**Plasma glucose** (mmol/L)	98.55 ± 3.12	100.19 ± 4.10	101.65 ± 2.95	89.79 ± 2.65 ***	0.001
**Insulin** (unit)	8.28 ± 1.33	8.12 ± 1.60	8.14 ± 1.03	6.58 ± 0.72 *	0.130
**HOMA-IR**	1.99 ± 0.34	1.88 ± 0.35	2.10 ± 0.28	1.46 ± 0.18 ***	0.024

Values are the mean ± SE. Placebo, starch; PT, pterocarpan-high soy leaf extract (2 g/day); FBG, fasting blood glucose; HbA_1_c, hemoglobin A_1_c; HOMA-IR, homeostatic model assessment of insulin resistance. * *p* < 0.05, *** *p* < 0.000 paired *t*-test between before and after trial in each group. ^†^ *p* < 0.05 general linear model for repeated measures ANOVA.

**Table 5:** Effect of pterocarpan-high soy leaf extract supplementation on changes of plasma AST and ALT in subjects with metabolic syndrome.

	Placebo		PT		Time × Group, *p* Value ^†^
	Before	After	Before	After	
**AST** (U·L^−1^)	10.03 ± 1.33	10.86 ± 1.63	9.79 ± 1.05	8.79 ± 0.74	0.158
**ALT** (U·L^−1^)	11.24 ± 0.56	11.34 ± 0.69	10.49 ± 0.52	10.13 ± 0.46	0.401

Values are the mean ± S.E. Placebo, starch; PT, pterocarpan-high soy leaf extract (2 g/day); AST, aspartate aminotransferase; ALT, alanine aminotransferase. ^†^ general linear model for repeated measures ANOVA.

**Table 6:** Effect of pterocarpan-high soy leaf extract supplementation on changes of plasma lipids concentrations in subjects with metabolic syndrome.

	Placebo		PT		Time × Group, *p* Value ^†^
	Before	After	Before	After	
**Triglyceride** (mg/dL)	187.19 ± 17.62	178.91 ± 14.99	176.41 ± 11.54	161.79 ± 13.13	0.243
**Free fatty acid** (mmol/L)	2.25 ± 0.08	1.98 ± 0.07 *	2.42 ± 0.15	1.60 ± 0.13 ***	0.000
**Total Cholesterol** (mg/dL)	165.54 ± 6.75	165.14 ± 6.43	173.00 ± 5.04	155.19 ± 7.80 *	0.077
**HDL-cholesterol** (mg/dL)	38.54 ± 2.87	26.34 ± 2.19	32.90 ± 2.27	36.38 ± 2.42	0.375
**non HDL-cholesterol** (mg/dL)	128.11 ± 7.70	132.68 ± 7.44	130.28 ± 10.74	108.76 ± 11.36 *	0.014
**LDL-cholesterol** (mg/dL)	91.77 ± 6.92	92.89 ± 6.68	107.24 ± 5.10	92.64 ± 7.80	0.801
**AI**	3.77 ± 0.38	3.81 ± 0.30	4.59 ± 0.36	3.81 ± 0.39	0.692

Values are the mean ± SE. Placebo, starch; PT, pterocarpan-high soy leaf extract (2 g/day); HDL, high density lipoprotein; LDL, low density lipoprotein; AI, atherogenic index. * *p* < 0.05, *** *p* < 0.000 paired *t*-test between before and after trial in each group. ^†^ *p* < 0.05 general linear model for repeated measures ANOVA.

**Table 7:** Effect of pterocarpan-high soy leaf extract supplementation on changes of plasma PAI-1, adipokine and cytokine levels in subjects with metabolic syndrome.

	Placebo		PT		Time × Group, *p* Value ^†^
	Before	After	Before	After	
**PAI-1** (ng/mL)	9.22 ± 0.47	10.28 ± 1.18	11.95 ± 1.01	10.18 ± 1.11 *	0.044
**TNF-α** (pg/mL)	12.86 ± 0.92	12.92 ± 0.72	12.71 ± 0.60	11.52 ± 0.51 *	0.031
**IL-6** (pg/mL)	6.14 ± 0.13	5.80 ± 0.11	6.51 ± 0.38	5.96 ± 0.21 *	0.872
**MCP-1** (pg/mL)	124.47 ± 13.66	131.52 ± 11.20	141.13 ± 10.00	126.72 ± 9.41	0.370
**Adiponectin** (ng/mL)	73.10 ± 10.55	65.92 ± 7.58	75.63 ± 8.29	71.06 ± 8.47	0.803
**Leptin** (pg/mL)	13.39 ± 1.66	14.16 ± 1.69	13.79 ± 1.55	13.40 ± 1.39	0.312

Values are the mean ± SE. Placebo, starch; PT, pterocarpan-high soy leaf extract (2 g/day); PAI-1, plasminogen activator inhibitor-1; TNF-α, tumor necrosis factor-α; MCP1, monocyte chemotactic protein 1; IL-6: interleukin-6. * *p* < 0.05 paired *t*-test between before and after trial in each group. ^†^ *p* < 0.05 general linear model for repeated measures ANOVA.

**Table 8:** KEGG pathway analysis of genes that were significantly regulated in pterocarpan-high soy leaf extract supplemented subjects.

KEGG Pathway	Downregulated Genes	Upregulated Genes
Cytokine–cytokine receptor interaction	*CCR4, CCR6*	
Chemokine signaling pathway	*CCR4, CCR6, LYN, RAP1B*	
NF-κB signaling pathway	*LYN, TRAF5, CCL4L2*	
Fatty acid metabolism	*ACSL4*	
PPAR signaling pathway	*ACSL4, PDPK1*	
AMPK signaling pathway	*PDPK1*	
Insulin signaling pathway	*PDPK1*	
FOXO signaling pathway	*PDPK1*	*ATG12*
Calcium signaling pathway	*ATP2A2, SLC25A6*	
Pathway in cancer	*TRAF5*	*LPAR5*
PI3K–AKT signaling pathway	*PDPK1*	*LPAR5*

Analyses were performed using KEGG pathway analysis (www.genome.jp/kegg) with genes that were differentially expressed pterocarpan-high extract, soy leaf extract supplemented subjects, compared with before pterocarpan-high soybean leaf extract supplementation. Genes up-, or downregulated in pterocarpan-high soybean leaf extract supplemented subjects compared with before pterocarpan-high soybean leaf extract supplementation; AMPK, AMP-activated protein kinase; KEGG, Kyoto Encyclopedia of Genes and Genomes; mTOR, mammalian target of rapamycin; TNF, tumor necrosis factor.

## Supplementary Materials

The following are available online at http://www.mdpi.com/2072-6643/8/11/734/s1.

## Abbreviations

PT, pterocarpan-high soybean leaf extract; PBMCs, peripheral blood mononuclear cells; BMI, body mass index; BP, blood pressure; WHR, waist-hip ratio; FBG, fasting blood glucose; HbA1c, hemoglobin A1c; HOMA-IR, homeostatic model assessment of insulin resistance; HDL, high density lipoprotein; LDL, low density lipoprotein; AI, atherogenic index; TNF-α, tumor necrosis factor alpha; IL-6, interleukin 6 monocyte; MCP-1, monocyte chemotactic protein-1; PAI, plasminogen activator inhibitor-1; HOMA-IR, homeostatic index of insulin resistance; PDPK-1, phosphoinositide-dependent protein kinase-1; CCR, CC chemokine receptor; CCL4L2, CC chemokine ligand 4-like 2.
