# Supplementary material for: Beneficial Effects of Pterocarpan-High Soybean Leaf Extract on Metabolic Syndrome in Overweight and Obese Korean Subjects: Randomized Controlled Trial
Source: Nutrients. 2016 Nov 18;8(11):734. doi: 10.3390/nu8110734 (PMC5133118; doi:10.3390/nu8110734)
Supplement: Supplementary file 1 [file nutrients-08-00734-s001.docx]

**Supplementary Materials: Beneficial Effects of Pterocarpan-High Soybean Leaf Extract on Metabolic Syndrome in Overweight and Obese Korean Subjects: Randomized Controlled Trial**

Ri Ryu, Tae-Sook Jeong, Ye Jin Kim, Ji-Young Choi, Su-Jung Cho, Eun-Young Kwon, Un Ju Jung, Hyeon-Seon Ji, Dong-Ha Shin and Myung-Sook Choi

## _
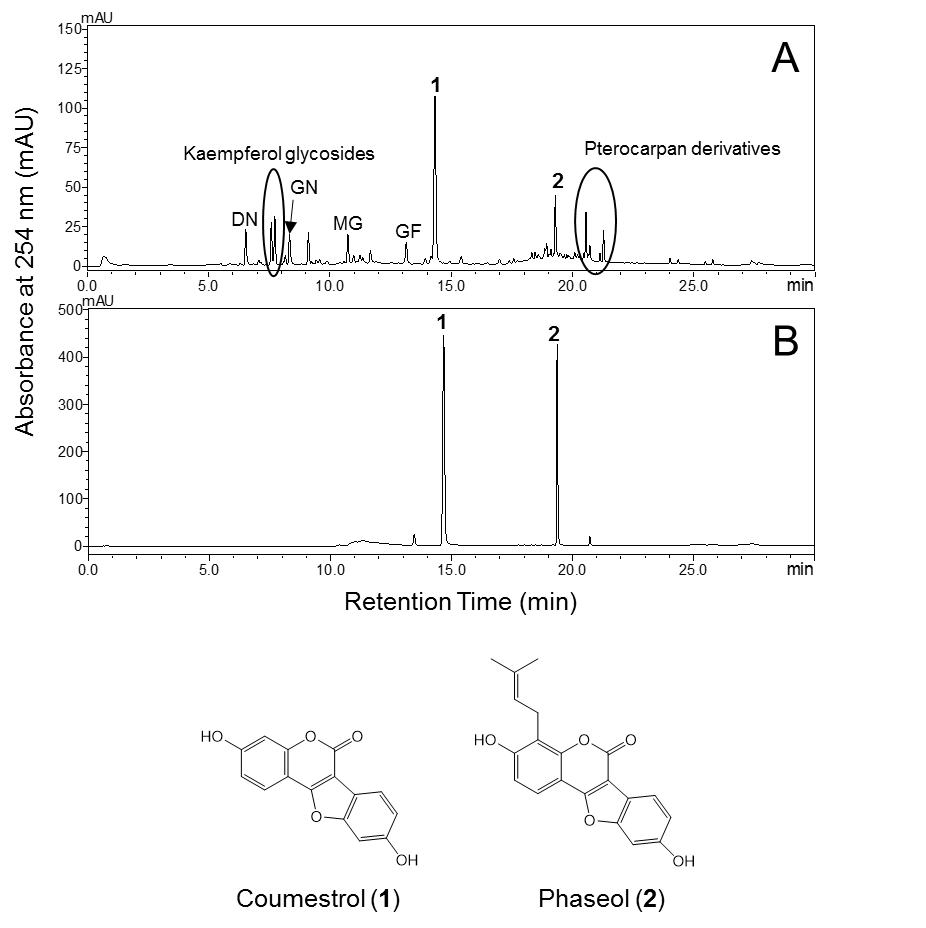
_

**Figure S1.** HPLC profiles of pterocarpan-high soybean leaf extract and the chemical structure of the main pterocarpan components—coumestrol and phaseol. HPLC chromatograms of (**A**) pterocarpan-high soybean leaf extract (PT) and (**B**) standard compounds—coumestrol (1) and phaseol (2)—were detected at 254 nm. DN, Daidzin; GN, genestin; MG, 6” 6”-O-malonylgenistin; GF; Glyceofuran.

**Table S1.** Total flavonoids and phenols contents analysis in PT.

| **Sample** | **Total Flavonoids  (mg Quercetin Equivalents/g Extract)** | **Total Phenolics  (mg Gallic Acid Equivalents/g Extract)** |
| --- | --- | --- |
| PT | 136.7 ± 0.0 | 77.3 ± 0.2 |
